# Supplementary material for: Ancient Species Diversity and Niche Adaptation in Tannerella and Porphyromonas Revealed Through Pangenomics
Source: Genome Biol Evol. 2026 Jun 8;18(6):evag136. doi: 10.1093/gbe/evag136 (PMC13298647; doi:10.1093/gbe/evag136)
Supplement: evag136_Supplementary_Data [file evag136_supplementary_data.zip › supplementary figures_revision_1_clean_accepted.pdf]

Supplementary tables and figures for:

Ancient species diversity and niche adaptation in *Tannerella* and *Porphyromonas* revealed through pangenomics

Authors: Aurore Galtier, Christina Warinner, Irina M. Velsko*

Tables (excel files)

Supplementary Table S1:
Supplementary Table S1. Metadata for *Tannerella* MAG and isolate genomes.

Supplementary Table S2:
Supplementary Table S2. Metadata for *Porphyromonas* MAG and isolate genomes.

Supplementary Table S3:
Supplementary Table S3. Virulence factors function and annotations.

Supplementary Table S4:
Supplementary Table S5. Genomes used as training files for Prokka annotations.

Supplementary Table S5:
Supplementary Table S4. *fimA* genotyping of MAGs.

Supplementary Table S6:
Supplementary Table S6. Pyseer summary table.

Figures

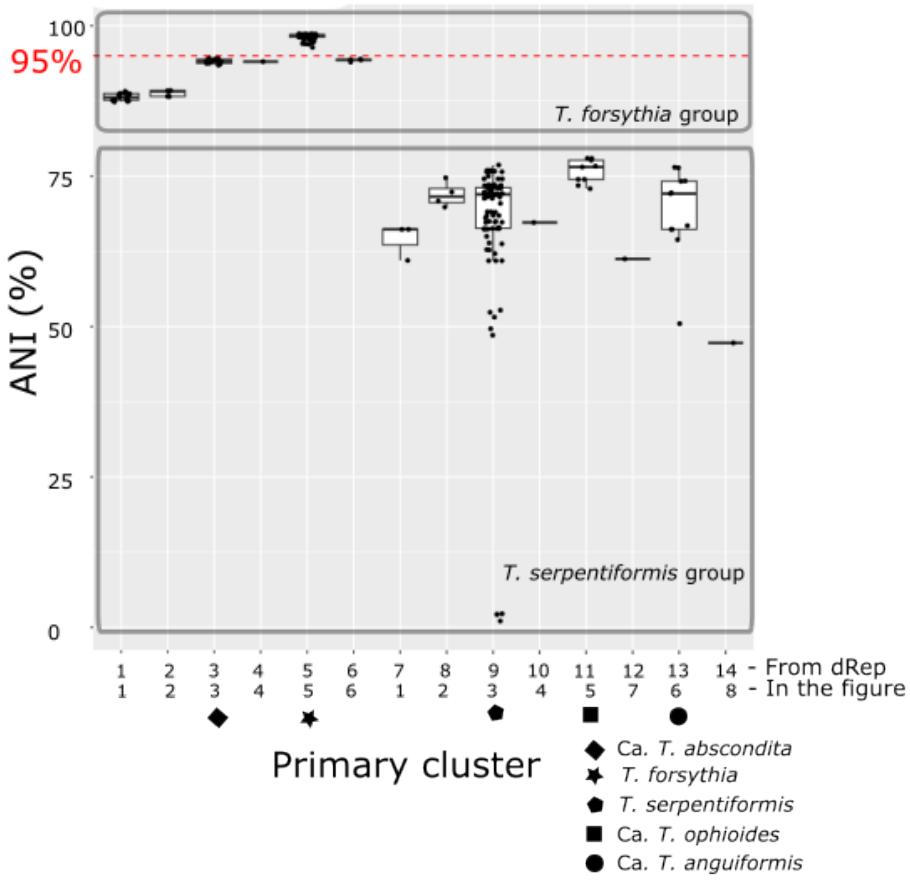

Supplementary Figure S1. Average nucleotide identity (ANI) between all *Tannerella* MAGs and the *Tannerella forsythia* MAGs.
MAGs with > 95% ANI (represented in the dashed red line) are considered to be a single species (in this figure *T. forsythia*), while MAGs falling below this cut-off are placed in independent clusters. The primary cluster numbers here correspond to those shown in the main text figures. See Supplementary table S1 to see the correspondence between the original cluster number generated by dRep and the updated main text cluster number.

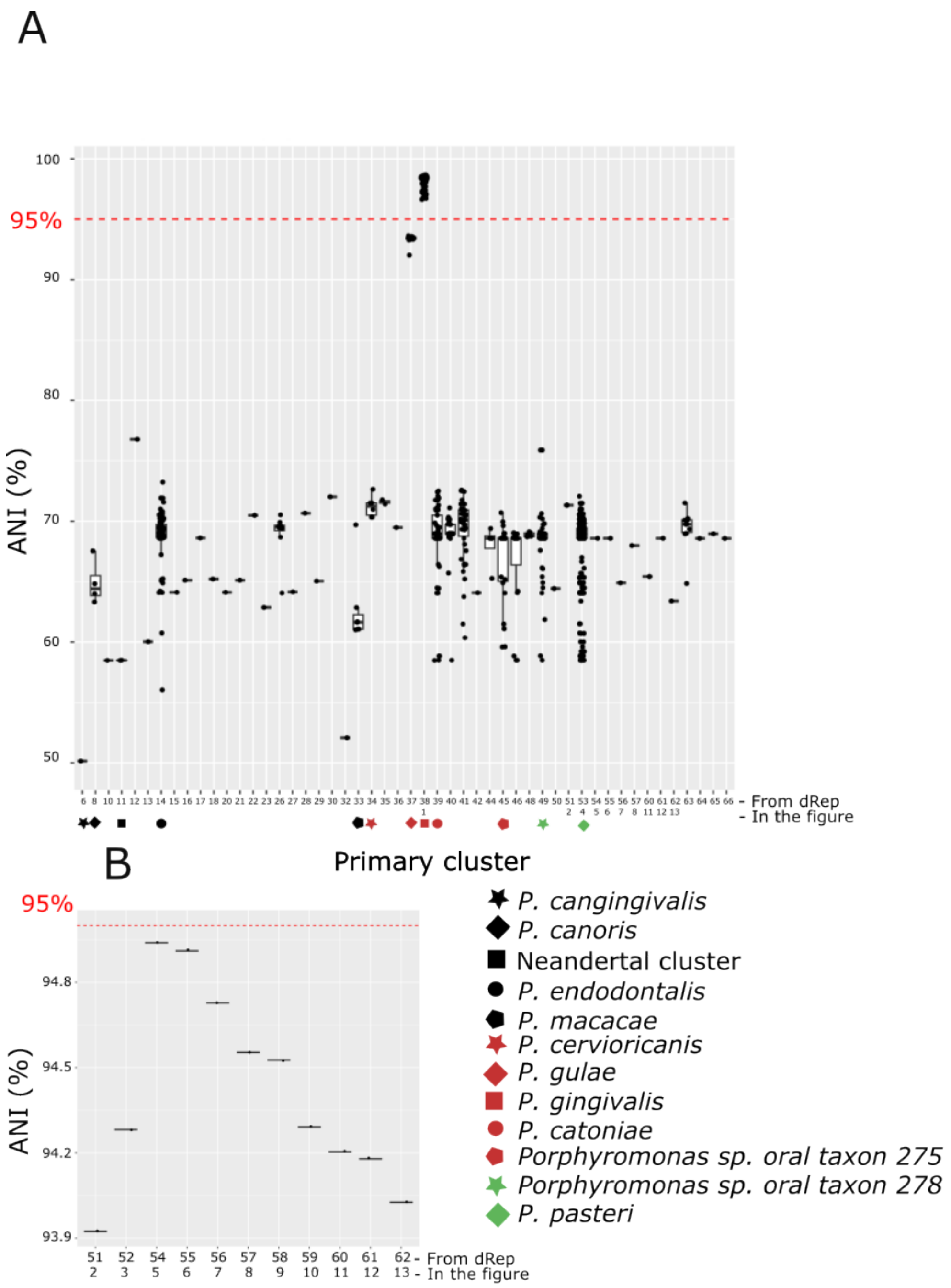

Supplementary Figure S2. Average nucleotide identity (ANI) between all *Porphyromonas* MAGs.

MAGs with > 95% ANI (represented in the dashed red line) are considered to be a single species (**A.** *P. gingivalis*; **B.** *P. pasteri*), while MAGs falling below this cut-off are placed in independent clusters. Only similarities > 50% are shown. The primary cluster numbers here correspond to those shown in the main text figures. See Supplementary Tables SX1 and SX2 to see the correspondence between the original cluster number generated by dRep and the updated main text cluster number. **A.** ANI of *Porphyromonas* MAGs in each cluster against the *P. gingivalis* cluster MAGs. The star indicates *P. pasteri*. **B.** All the *P. pasteri* cluster MAGs against the *P. pasteri* unique primary cluster. These MAGs falling outside the 95% ANI cut-off may have lower completeness/contamination that prevents them from reaching 95% ANI with other *P. pasteri* MAGs. Colors are used to distinguish repeated shapes, and do not correspond to clusters or groups.

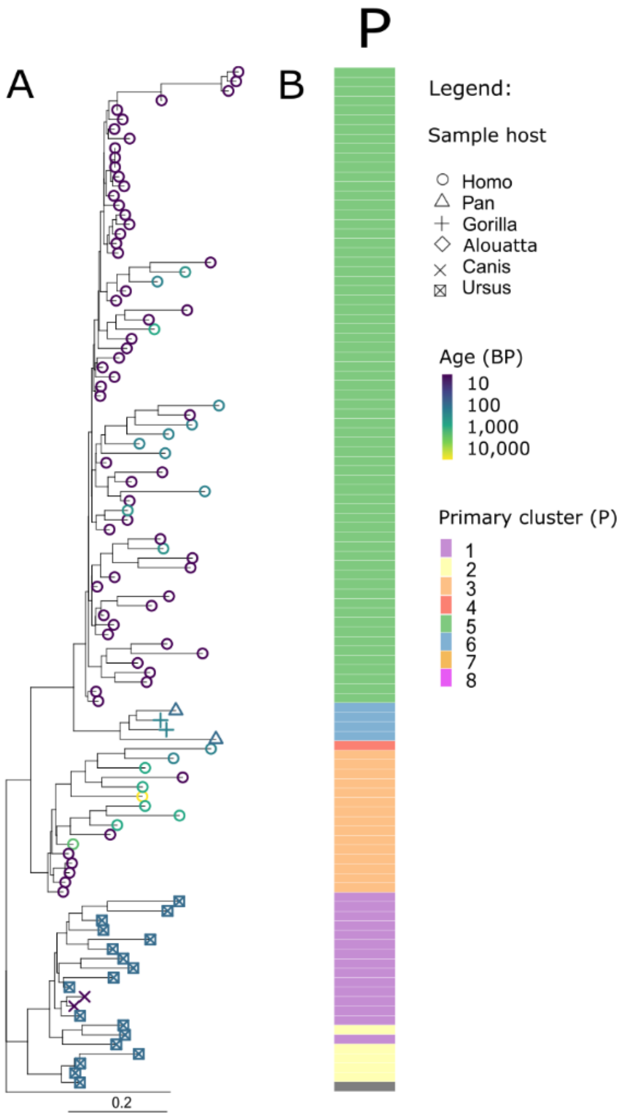

Supplementary Figure S3. Phylogenetic relationship of *Tannerella forsythia* cluster MAGs using 400 universal genes from PhyloPhlan to confirm the tree structure. The tree tip color corresponds to the sample's age in years before present (BP), and the tip shape to the sample host. The matrix gives information about the species cluster (based on 95% ANI cut-offs). The metadata matrix displays the species clusters with an average nucleotide identity (ANI) of ≥ 95%. The scale bar indicates substitution per position. *Tannerella serpentiformis* was used as the outgroup to root the tree. **A.** Maximum-likelihood tree. **B.** Primary cluster metadata matrix.

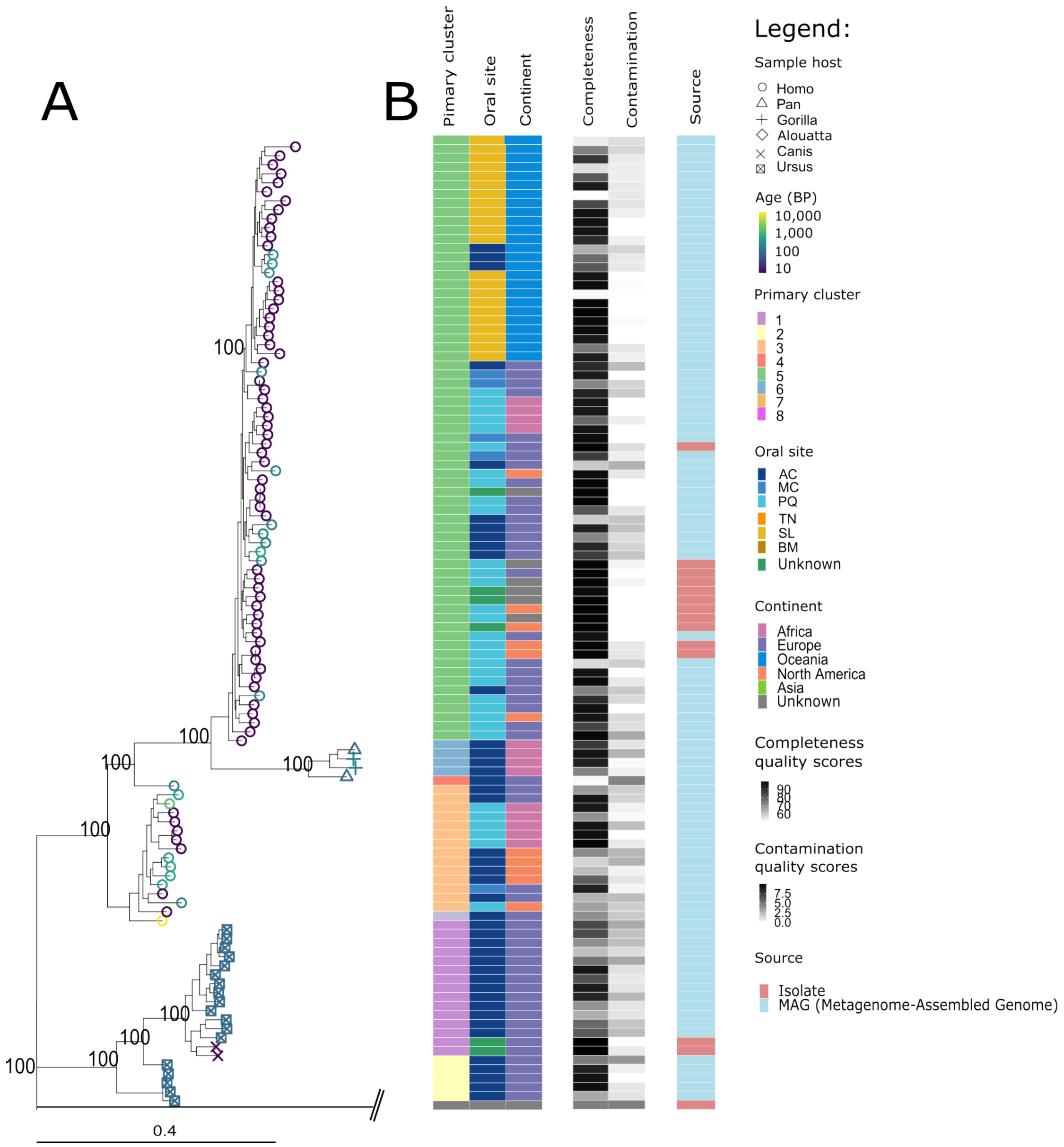

Supplementary Figure S4. Phylogenetic relationship of *Tannerella forsythia* MAGs to the sample and MAG metadata.

The tree tip color corresponds to the sample's age in years before present (BP), and the tip shape to the sample host. The matrix gives information about the species cluster (based on 95% ANI cut-offs), oral site, continent, and original publication of the samples from which MAGs are assembled, and completeness and contamination estimates of the MAGs. Bootstrap support based on 100 replicates is indicated as percentages for principal branches. The scale bar indicates substitution per position. *Tannerella serpentiformis* was used as the outgroup to root the tree. **A.** Maximum-likelihood phylogenetic tree of *Tannerella forsythia* group (same as main Figure 2A). **B**. Metadata matrix of *Tannerella forsythia* group. Oral site - Oral: The sampling site was not specified. AC: Ancient calculus, MC: modern calculus, PQ: plaque, TN: tongue, SL: saliva, BM: buccal mucosa, HP: hard palate.

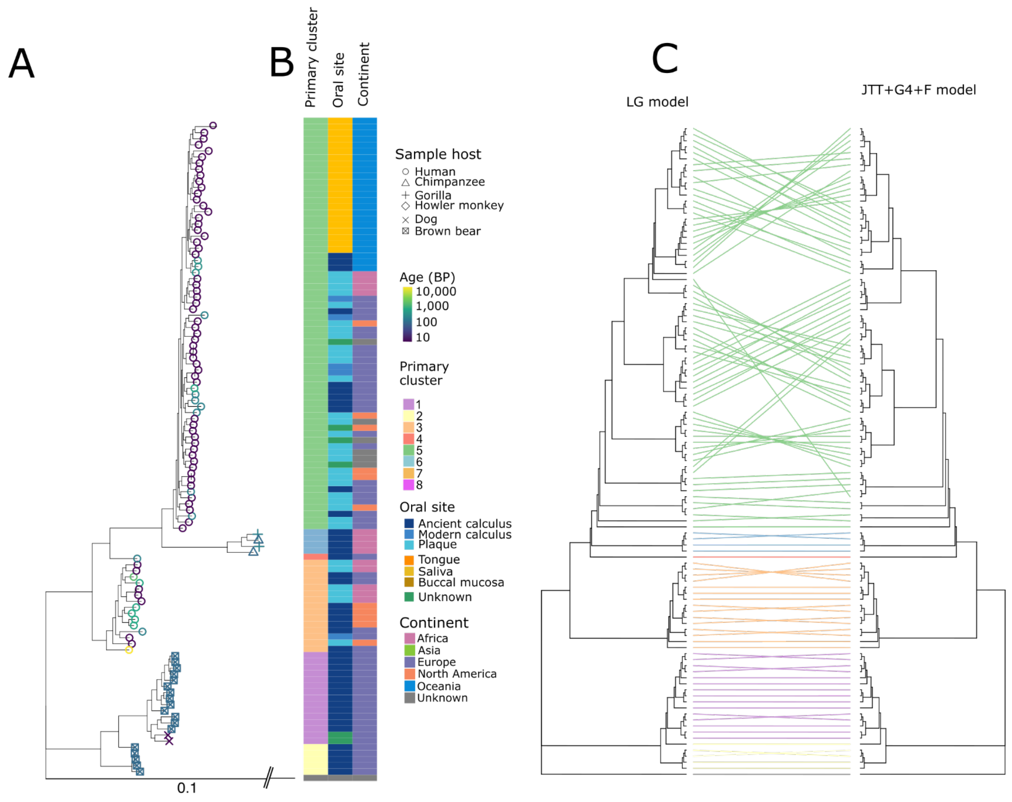

Supplementary Figure S5. Phylogenetic relationship of *Tannerella forsythia* group MAGs to the sample and MAG metadata using the JTT+G4+F model and tanglegram comparing the two trees.

The tree tip color corresponds to the sample's age in years before present (BP), and the tip shape to the sample host. The scale bar indicates substitution per position. *Tannerella serpentiformis* was used as the outgroup to root the tree. **A.** Maximum-likelihood phylogenetic tree of the *Tannerella forsythia* group built with RAxML using the JTT+G4+F model, which was determined to be the optimal substitution model by modeltest-ng. **B**. Metadata matrix of *Tannerella forsythia* group, same information as shown in main text Figure 2b. **C.** Tanglegram comparing the *T. forsythia* group tree built with the LG substitution model (same as main text Figure 2a) and the JTT+G4+F model tree from panel A here. Lines connect the corresponding MAG/genome in each tree and are colored by MAG/genome.

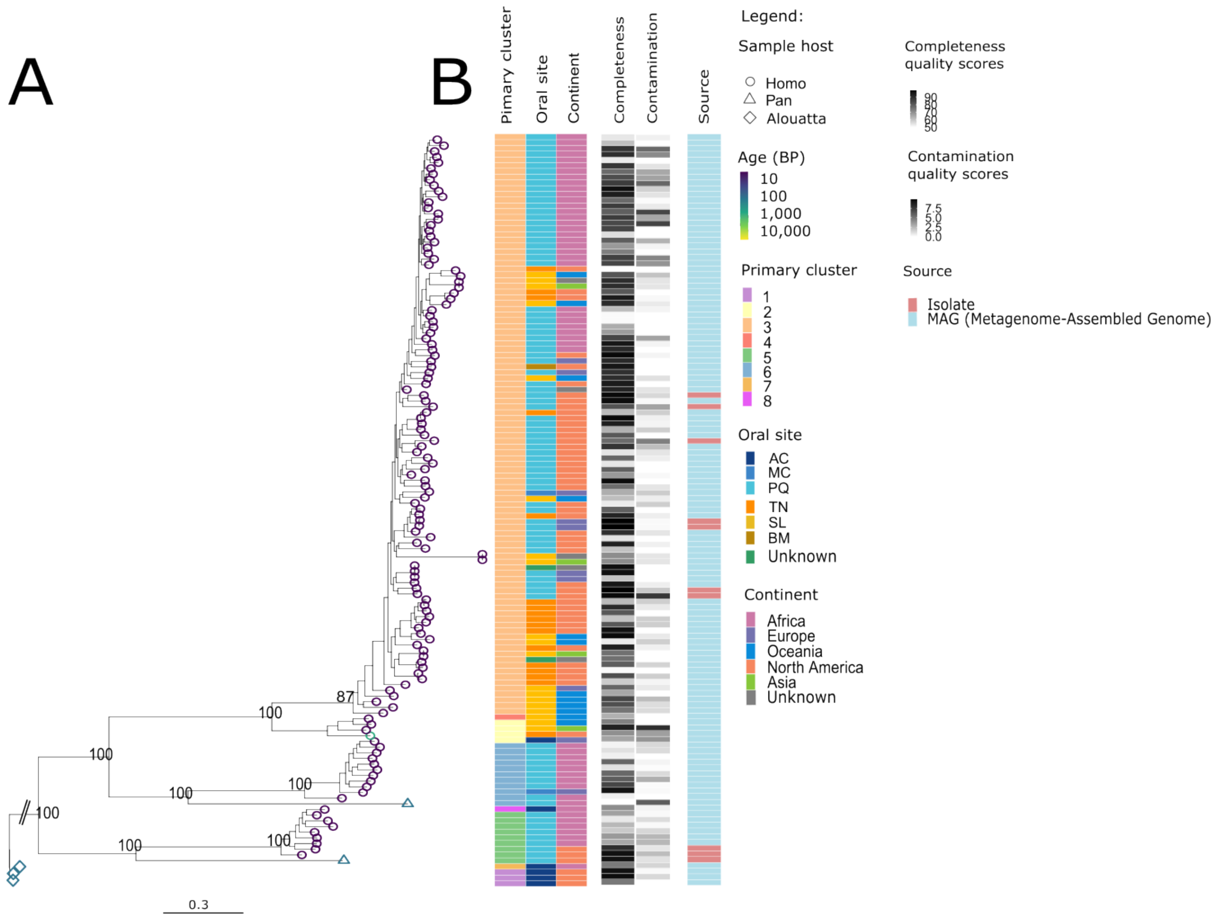

Supplementary Figure S6. Phylogenetic relationship of *Tannerella serpentiformis* MAGs to the sample and MAG metadata.

The tree tip color corresponds to the sample's age in years before present (BP), and the tip shape to the sample host. The metadata matrix gives information about the species cluster (based on 95% ANI cut-offs), oral site, continent, and original publication of the samples from which MAGs are assembled, as well as completeness and contamination estimates of the MAGs. Bootstrap support values based on 100 replicates are indicated as percentages for principal branches. The scale bar indicates substitutions per position. The tree was rooted using a MAG from a howler monkey (*Alouatta*) sample. **A.** Maximum-likelihood phylogenetic tree of *Tannerella serpentiformis* group. **B.** Metadata matrix of *Tannerella serpentiformis* group. Oral site - Oral: The sampling site was not specified. AC: Ancient calculus, MC: modern calculus, PQ: plaque, TN: tongue, SL: saliva, BM: buccal mucosa, HP: hard palate.

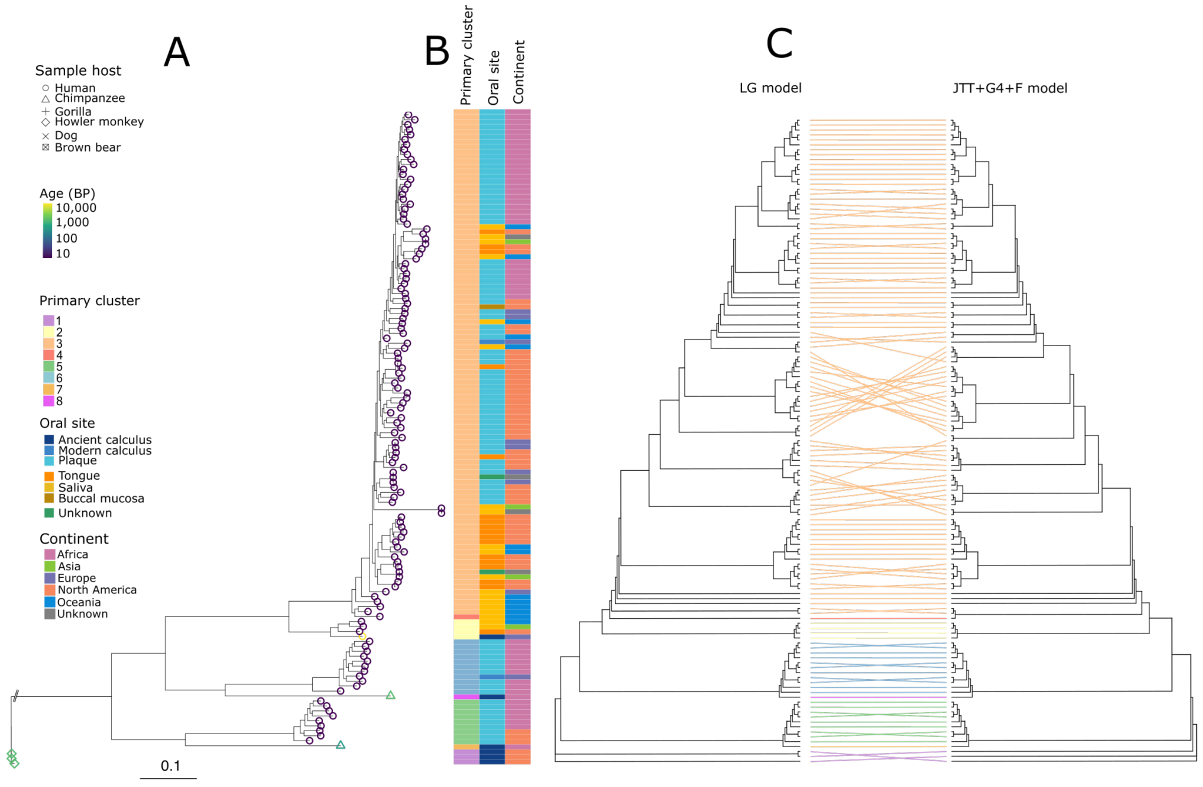

Supplementary Figure S7. Phylogenetic relationship of *Tannerella serpentiformis* group MAGs to the sample and MAG metadata using the JTT+G4+F model and tanglegram comparing the two trees.

The tree tip color corresponds to the sample's age in years before present (BP), and the tip shape to the sample host. The scale bar indicates substitution per position. The tree was rooted using a MAG from a howler monkey (*Alouatta*) sample. **A.** Maximum-likelihood phylogenetic tree of *Tannerella serpentiformis* group built with RAxML using the JTT+G4+F model, which was determined to be the optimal substitution model by modeltest-ng. **B**. Metadata matrix of *Tannerella serpentiformis* group, same information as shown in main text Figure 2d. **C.** Tanglegram comparing the *T. serpentiformis* group tree built with the LG substitution model (same as main text Figure 2c) and the JTT+G4+F model tree from panel A here. Lines connect the corresponding MAG/genome in each tree and are colored by MAG/genome.

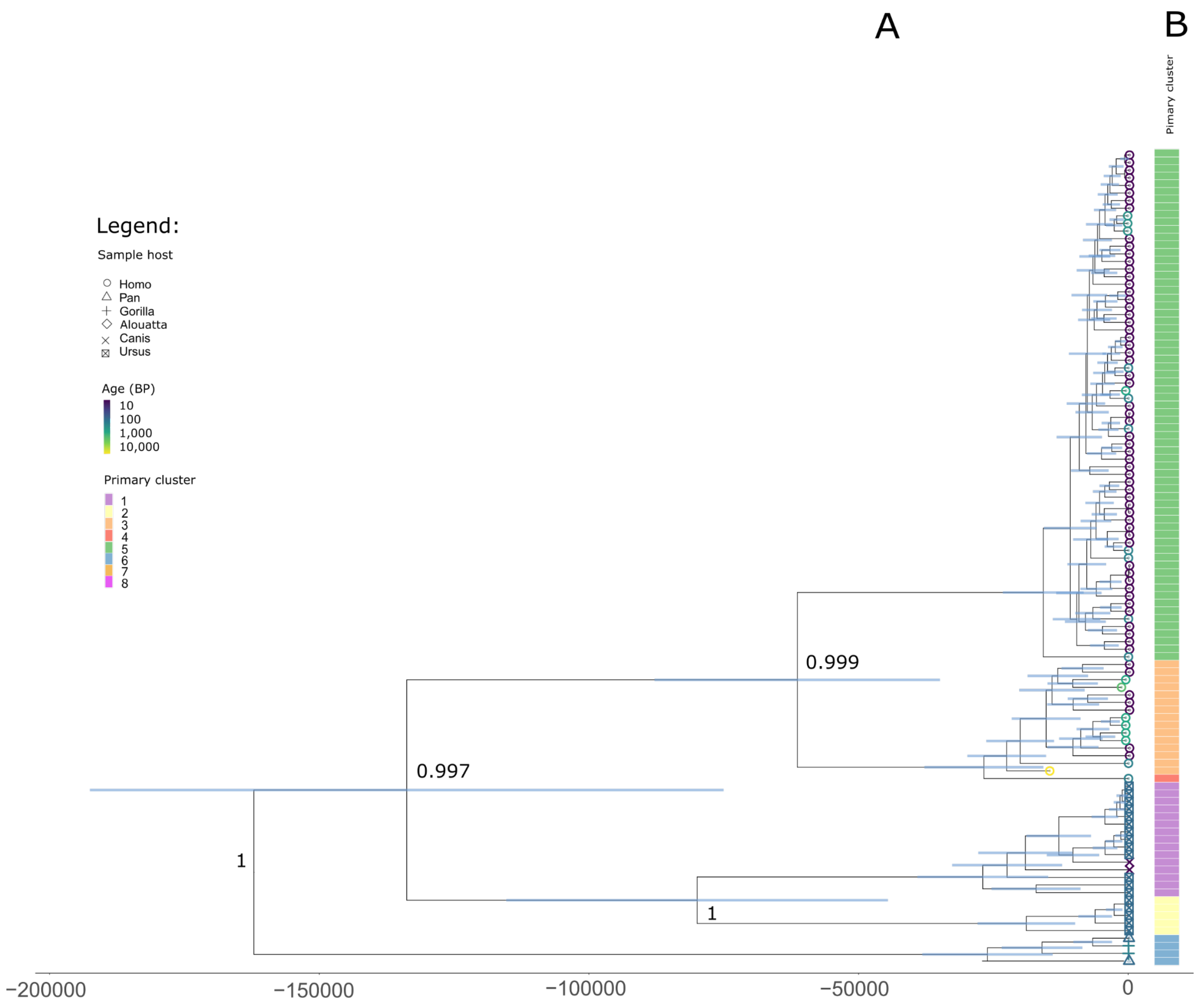

Supplementary Figure S8: BEAST analysis to estimate the split time between *T. forsythia* and Ca. *T. abscondita*.

Tree tip color corresponds to sample age in years before present (BP), and the tip shape to the sample host. The metadata matrix shows the 95% ANI-based species cluster. Posterior probabilities are indicated for principal branches. The blue bars indicate the 95% confidence interval of the age estimates. **A**. Maximum likelihood BEAST tree of *Tannerella forsythia* group. **B**. Metadata matrix of *Tannerella forsythia* group.

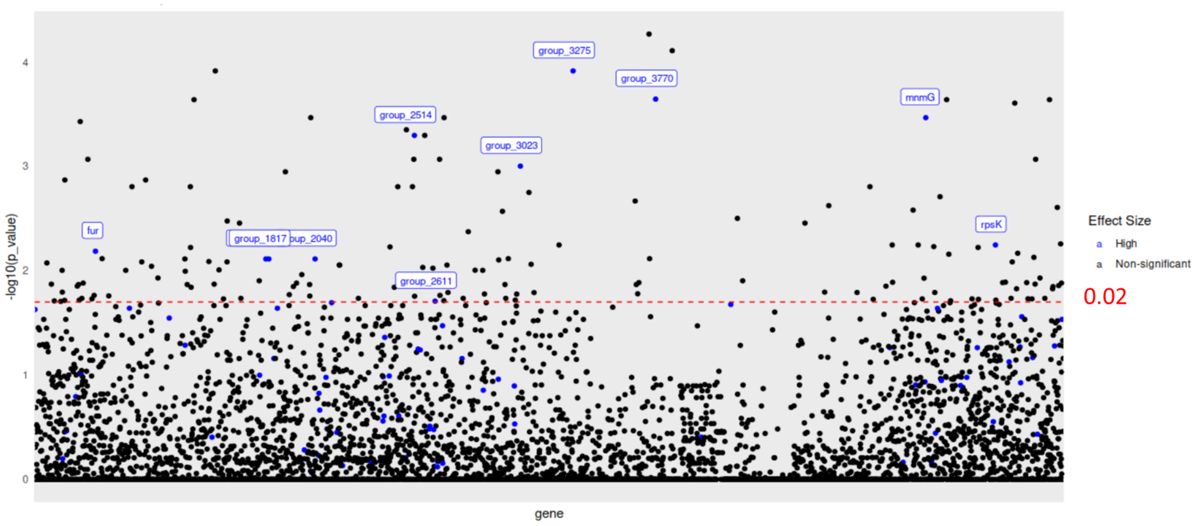

Supplementary Figure S9. Gene association with sample geographic origin.
Gene Manhattan plot from pyseer. Analysis used a fixed model, with p-values corrected for population structure and for false positives using the Benjamini-Hochberg correction. Dots are labeled and colored blue if the p-value is less than 0.02 and the effect size is high (>0.5), indicating a significant association with the continent of origin of the sample. All the MAGs are from the *Tannerella forsythia* clade; 25 Oceanian MAGs are compared to 85 MAGs/reference genomes from other parts of the world.

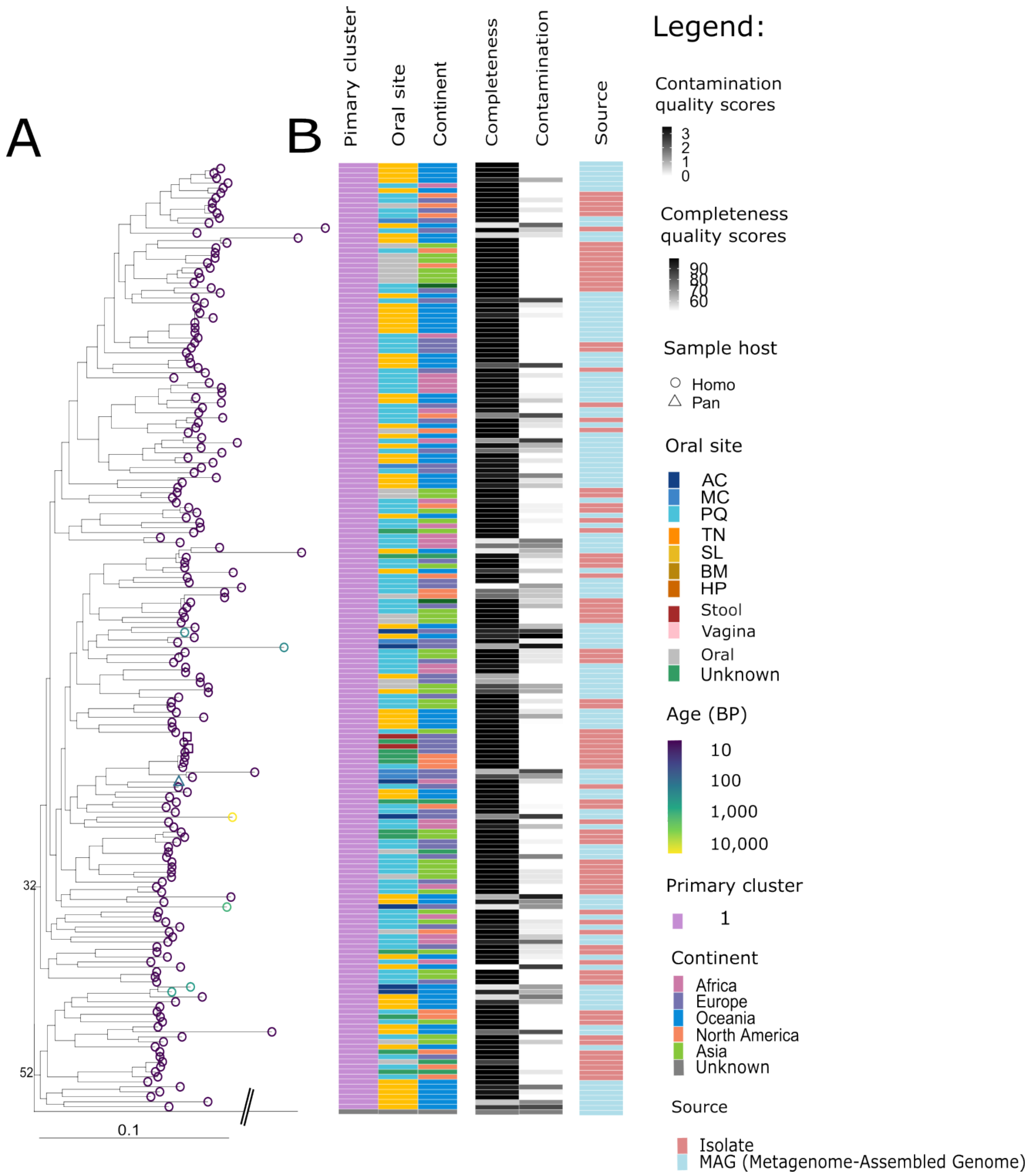

Supplementary Figure S10. Phylogenetic relationship of *Porphyromonas gingivalis* to geographic origin, oral sample site, studies, completeness, and contamination estimates of MAGs.

The tree tip color corresponds to the sample's age in years before present (BP), and the tip shape to the sample host. The matrix gives information about the species cluster (based on 95% ANI cut-offs), oral site, continent, and original publication of the samples from which MAGs are assembled, and completeness and contamination estimates of the MAGs. The tree was rooted using a *Porphyromonas pasteri* reference genome from NCBI. **A**. Maximum-likelihood phylogenetic tree of *Porphyromonas gingivalis* species cluster. **B**. Metadata matrix of *Porphyromonas gingivalis*. Oral site - Oral: The sampling site was not specified. AC: Ancient calculus, MC: modern calculus, PQ: plaque, TN: tongue, SL: saliva, BM: buccal mucosa, HP: hard palate.

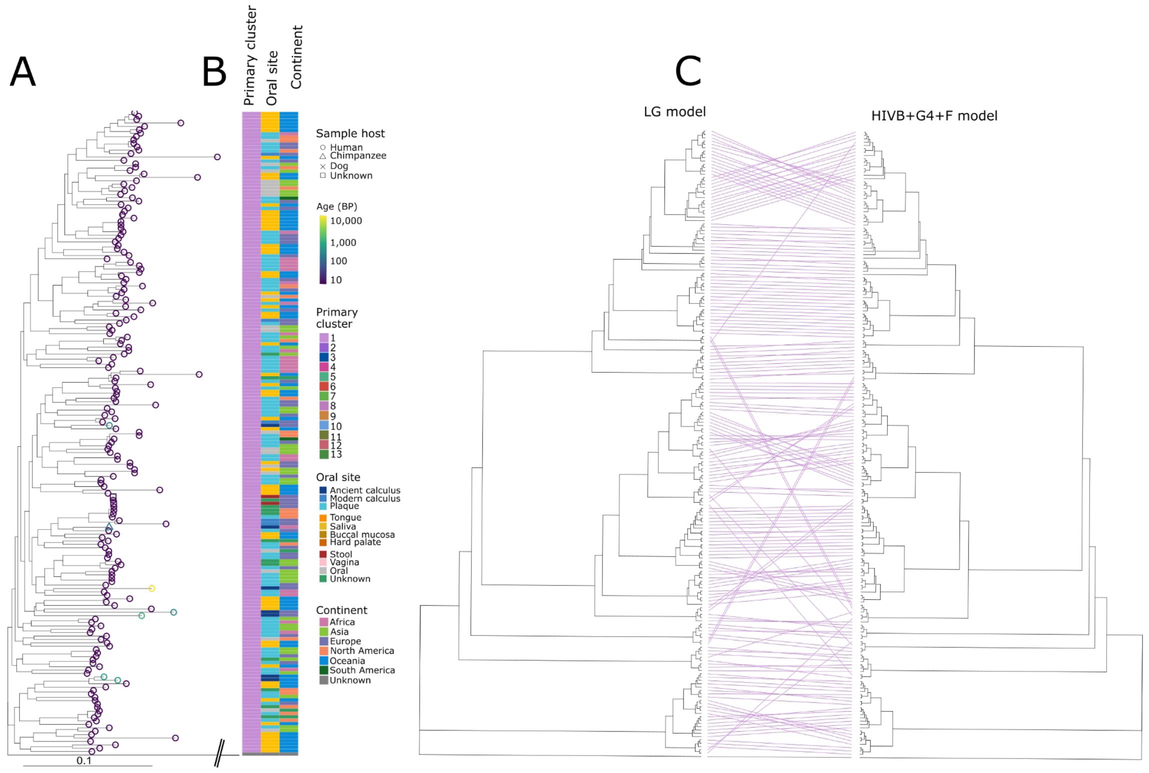

Supplementary Figure S11. Phylogenetic relationship of *Porphyromonas gingivalis* MAGs to the sample and MAG metadata using the HIVB+G4+F model and tanglegram comparing the two trees.

The tree tip color corresponds to the sample's age in years before present (BP), and the tip shape to the sample host. The scale bar indicates substitution per position. The tree was rooted using a *Porphyromonas pasteri* reference genome from NCBI. **A.** Maximum-likelihood phylogenetic tree of *Porphyromonas gingivalis* built with RAxML using the HIVB+G4+F model, which was determined to be the optimal substitution model by modeltest-ng. **B**. Metadata matrix of *Porphyromonas gingivalis*, same information as shown in main text Figure 4b. **C.** Tanglegram comparing the *P. gingivalis* tree built with the LG substitution model (same as main text Figure 4a) and the HIVB+G4+F model tree from panel A here. Lines connect the corresponding MAG/genome in each tree and are colored by MAG/genome.

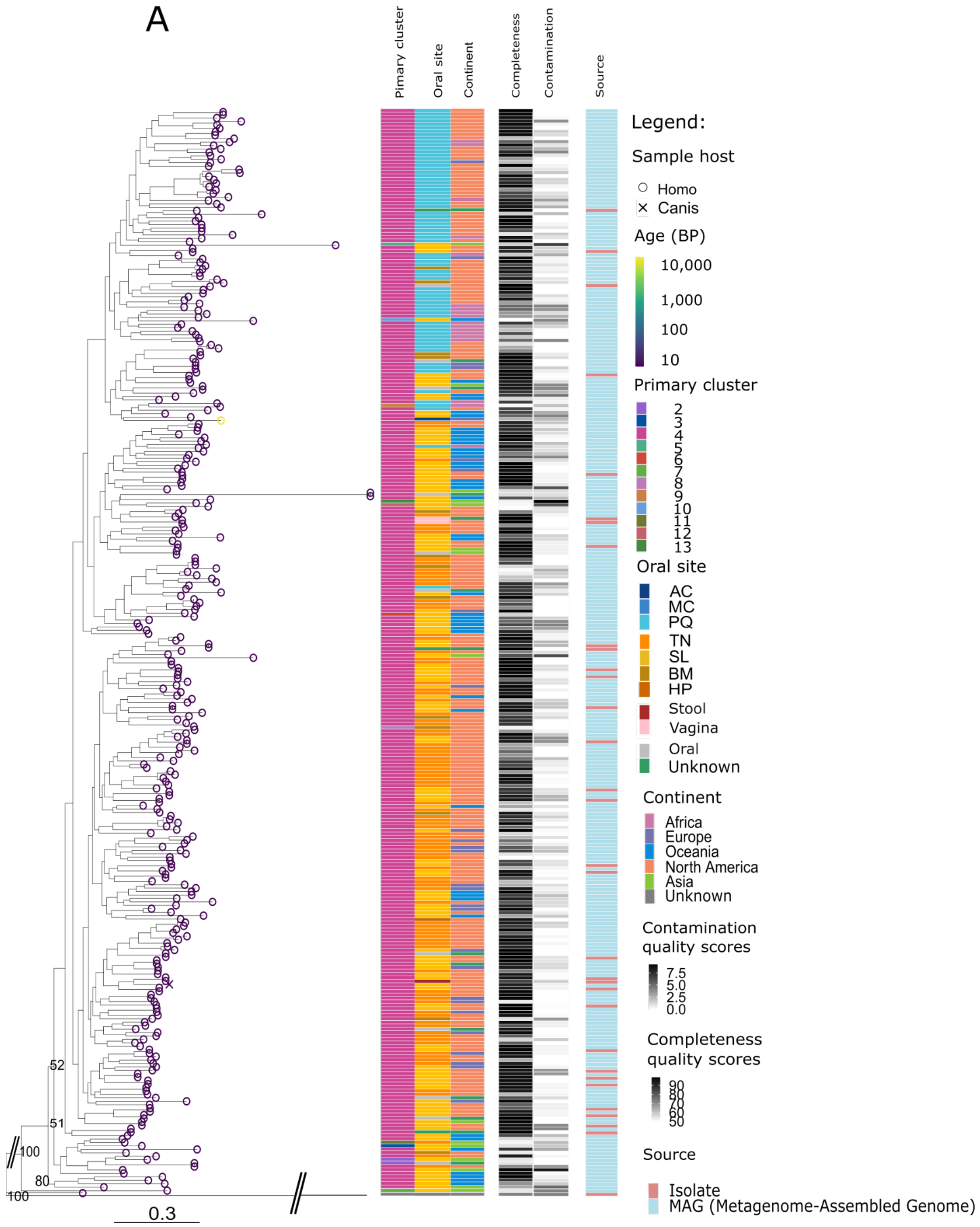

Supplementary Figure S12. Phylogenetic relationship of *Porphyromonas pasteri* to geographic origin, oral sample site, studies, completeness, and contamination estimates of MAGs.

The tree tip color corresponds to the sample's age in BP (years Before Present), and the tip shape to the sample host. The matrix gives information about the species cluster (based on 95% ANI cut-offs), oral site, continent, and original publication of the samples from which MAGs are assembled, and completeness and contamination estimates of the MAGs. The tree was rooted using the midpoint option in R. A. Maximum-likelihood phylogenetic tree of *Porphyromonas pasteri* species cluster. B. Metadata matrix. Oral site - Oral: The sampling site was not specified. AC: Ancient calculus, MC: modern calculus, PQ: plaque, TN: tongue, SL: saliva, BM: buccal mucosa, HP: hard palate.

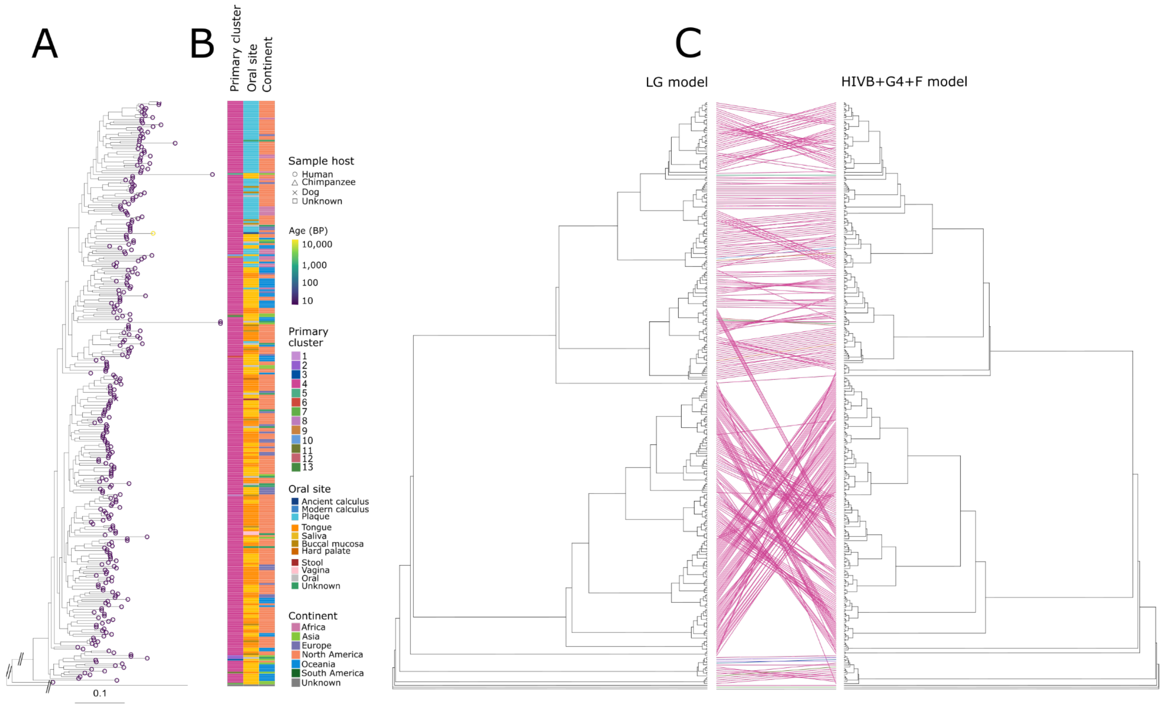

Supplementary Figure S13. Phylogenetic relationship of *Porphyromonas pasteri* MAGs to the sample and MAG metadata using the best model and tanglegram comparing the two trees.

The tree tip color corresponds to the sample's age in years before present (BP), and the tip shape to the sample host. The scale bar indicates substitution per position. The tree was rooted using the midpoint option in R, with *P. gingivalis* included as an outgroup. **A.** Maximum-likelihood phylogenetic tree of *Porphyromonas pasteri* group built with RAxML using the JTT+G4+F model, which was determined to be the optimal substitution model by modeltest-ng. **B**. Metadata matrix of *Porphyromonas pasteri*, same information as shown in main text Figure 4d. **C.** Tanglegram comparing the *P. pasteri* tree built with the LG substitution model (same as main text Figure 2c) and the JTT+G4+F model tree from panel A here. Lines connect the corresponding MAG/genome in each tree and are colored by MAG/genome.

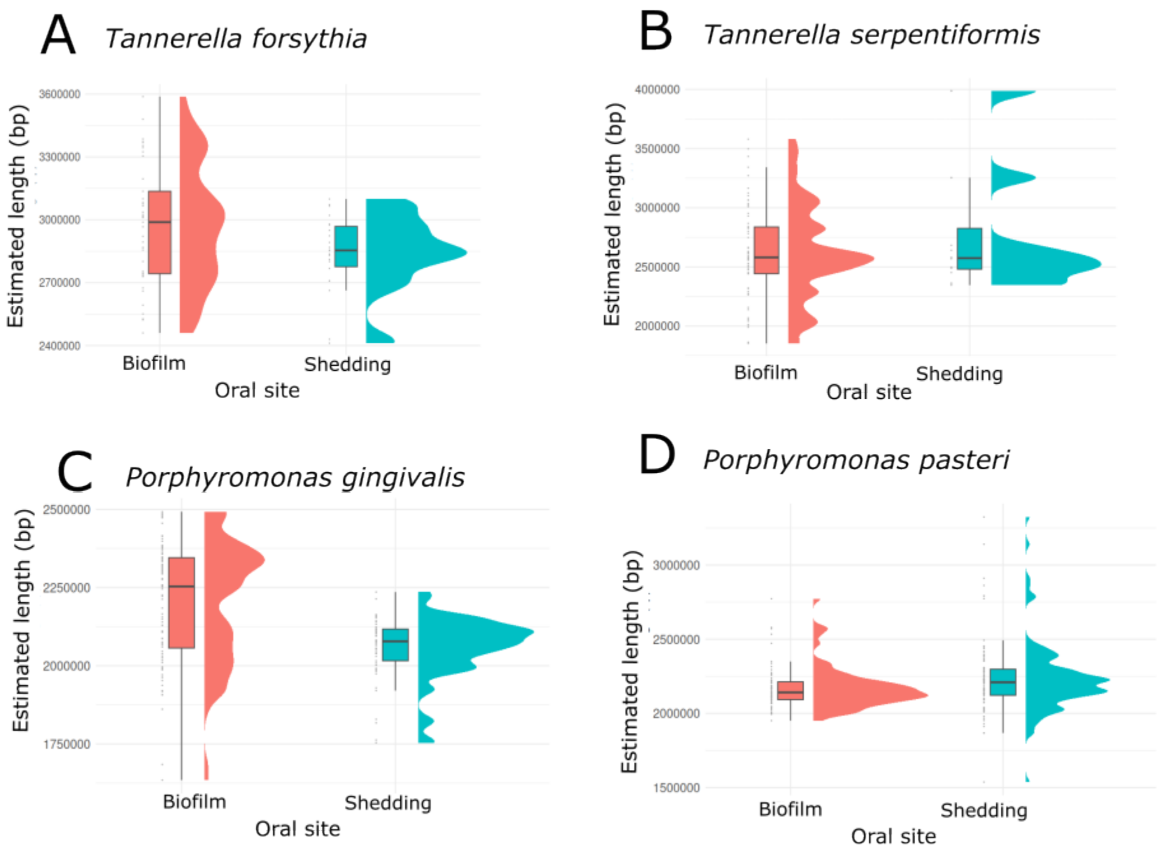

Supplementary Figure S14. Genome length comparison between shedding and non-shedding biofilm surfaces.

The length is estimated based on the completeness of the MAGs and the length of the corresponding reference genome from NCBI RefSeq. **A.** *T. forsythia*. **B**. *T. serpentiformis*. **C**. *P. gingivalis*. **D**. *P. pasteri.* No significant differences were detected between MAGs from biofilms vs. shedding surfaces. Shedding surfaces include saliva, tongue, buccal mucosa, and hard palette; non-shedding biofilm surfaces include dental plaque and calculus (ancient and modern).

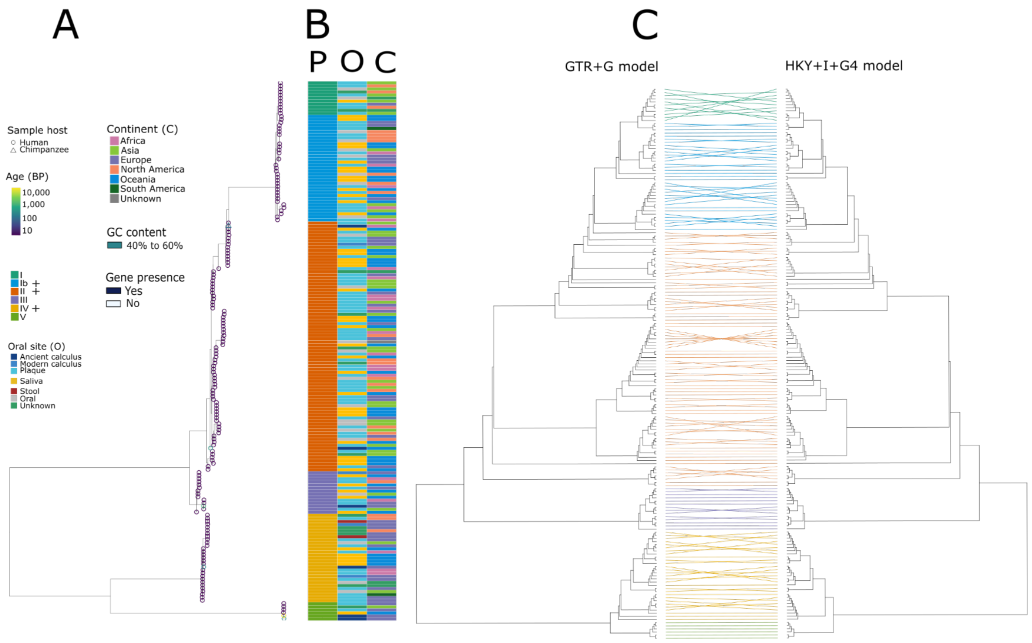

Supplementary Figure S15. Phylogenetic relationship of *fimA* and metadata information of the associated MAGs.

A. Phylogenetic tree of *fimA* gene sequences from *P. gingivalis*, built using RAxML and the HKY+I+G4 substitution model. The tree tip color corresponds to the age of the samples in years before present (BP) and shape corresponds to the sample host. The scale bar indicates substitutions per position. A (+) by the genotype indicates that the genotype is strongly associated with periodontal disease. **B.** Metadata matrix of the *fimA* genotype, primary cluster (P), oral site (O), and geographic localization (C) of samples that produced *P. gingivalis* MAGs, showing the same information as in main text Figure 5b. **C.** Tanglegram comparing the FimA tree built with the GTR+G substitution model (same as main text Figure 5a) and the HKY+I+G4 model tree from panel A here. Lines connect the corresponding MAG/genome in each tree and are colored by MAG/genome.
